# Relationship between Motor Symptoms, Cognition, and Demographic Characteristics in Treated Mild/Moderate Parkinson’s Disease

**DOI:** 10.1371/journal.pone.0123231

**Published:** 2015-04-23

**Authors:** Jay S. Schneider, Stephanie Sendek, Chengwu Yang

**Affiliations:** 1 Dept. of Pathology, Anatomy and Cell Biology, Thomas Jefferson University, Philadelphia, PA, United States of America; 2 Department of Public Health Sciences, College of Medicine, The Pennsylvania State University, Hershey, PA, United States of America; University of California at San Francisco, UNITED STATES

## Abstract

**Background:**

Although Parkinson’s disease (PD) is a progressive neurodegenerative disorder characterized primarily by motor symptoms, PD patients, at all stages of the disease, can experience cognitive dysfunction. However, the relationships between cognitive and motor symptoms and specific demographic characteristics are not well defined, particularly for patients who have progressed to requiring dopaminergic medication.

**Objective:**

To examine relationships between motor and cognitive symptoms and various demographic factors in mild to moderate, PD patients requiring anti-PD medication.

**Methods:**

Cognitive function was assessed in 94 subjects with a variety of neuropsychological tests during baseline evaluations as part of an experimental treatment study. Data were analyzed in relation to Unified Parkinson’s Disease Rating Scale motor scores and demographic variables.

**Results:**

Of the UPDRS subscores analyzed, posture/balance/gait was associated with the highest number of adverse cognitive outcomes followed by speech/facial expression, bradykinesia, and rigidity. No associations were detected between any of the cognitive performance measures and tremor. Motor functioning assessed in the “off” condition correlated primarily with disease duration; neuropsychological performance in general was primarily related to age.

**Conclusion:**

In PD patients who have advanced to requiring anti-PD therapies, there are salient associations between axial signs and cognitive performance and in particular, with different aspects of visuospatial function suggesting involvement of similar circuits in these functions. Associations between executive functions and bradykinesia also suggest involvement similar circuits in these functions.

## Introduction

Parkinson’s disease (PD) is a progressive neurodegenerative disorder characterized primarily by motor symptoms including bradykinesia, resting tremor, rigidity, and postural instability. However, it has become increasingly apparent that PD patients, at all stages of the disease, can experience cognitive dysfunction. Although there is heterogeneity to the clinical presentation of cognitive impairment in PD in general [1] and particularly earlier in the disease process, the cognitive impairment in PD often involves executive function, attention, visuospatial function and working memory, suggesting a frontal or fronto-striatal nature to these cognitive deficits [2,3,4,5].

Several studies have examined relationships between motor symptoms and cognitive functions in PD and in general, such studies have reported that bradykinesia and rigidity are correlated with at least some types of cognitive impairment while tremor is less likely to correlate with cognitive impairment [6,7,8,9]. For example, Huber et al. [10] compared levodopa-treated patients (Hoehn and Yahr stage 2–3) with tremor predominant disease to those with bradykinesia/rigidity dominant disease and found only the latter group to exhibit memory, visuospatial, and executive function deficits. Further, they found that age of onset of PD had little influence on neuropsychological performance when overall effect of age was controlled for [10]. However, these and similar findings are difficult to interpret since patients were tested while medicated and the effects of dopaminergic medications on cognition are varied and may affect individual cognitive symptoms differentially [11].

To overcome this potential problem with the interpretation of data obtained from medicated patients, other studies examining the relationship between motor symptoms and cognition in PD have utilized newly diagnosed, drug naive (de novo) patients [12,13]. In one such study, bradykinesia was associated with working memory and mental flexibility, whereas axial signs were associated with episodic memory and visuospatial function [13]. These authors suggested that slow movement and inflexible thinking may be controlled by a dopaminergic network different from networks involved in tremor and/or rigidity, since after controlling for age, sex, and education, no association was found between cognitive performance and tremor and rigidity [13]. However, a caveat of these studies is that cognitive deficits may be subtle in newly diagnosed, drug naive PD patients [14,15] and it may be difficult to relate cognitive functioning to other variables in this population, depending in large part on what types of cognitive assessments are performed and how sensitive they are to detecting subtle cognitive deficits in this population.

Although there are obvious merits to examining the relationship between cognitive and motor symptoms in de novo PD patients without having to account for the potential complicating factor of medication effects, it is also important to examine these relationships in more advanced patients who require medication as the relationship between cognitive and motor symptoms may change as the disease progresses. The purpose of the current study was to examine the relationship between motor and cognitive symptoms and various demographic factors in mild to moderate, PD patients requiring anti-PD medication and examined in a practically defined off period.

## Materials and Methods

### Participants and Ethics Statement

These data were collected during baseline assessments performed as part of a study examining long-term effects of GM1 ganglioside treatment on PD (ClinicalTrials.gov NCT00037830). The study was approved by the Division of Human Subjects Protection at Thomas Jefferson University and written informed consent was obtained from all subjects prior to enrollment. Subjects were men or women between 39 and 85 years of age with a diagnosis of idiopathic PD consistent with the UK PD Society brain bank PD diagnostic criteria. Inclusion criteria included “off” Unified Parkinson’s Disease Rating Scale (UPDRS) motor score between 10 and 40, “off” Hoehn and Yahr staging of 1.0 to 3.0, Mini Mental State Exam (MMSE) score ≥ 25, and Beck Depression Inventory (BDI-II) score ≤ 10.

### Procedures and Data Collection

All subjects were assessed while in a practically defined “off” period prior to taking that day's first dose of anti-PD medication and at least 12 h after the last dose of levodopa (or 24 h after the last dose of dopamine agonist). Pre-morbid full scale IQ (FS IQ) was estimated using the National Adult Reading Test (NART). Motor function was assessed by two independent observers, using the UPDRS part III (motor score). The motor score used in the analysis was the average score from the two observers. Inter-rater reliability was assessed using Pearson linear correlation coefficient for each pair of raters and inter-rater reliability was high (median correlation coefficient was 0.94 for UPDRS motor scores). Additionally, a rigidity sum was computed as the total of UPDRS items 22a-e; a bradykinesia sum was computed as the total of UPDRS item numbers 23a-b, 24a-b, 25a-b, 26a-b, and 31; an axial sum (posture/balance/gait) was computed as the total of UPDRS item numbers 27–30; a tremor sum was computed as the total of UPDRS item numbers 20a-e and 21a-b; a speech and facial expression sum was computed as the total of UPDRS item numbers 18 and 19.

The following tests were used to assess neuropsychological functions:

#### Brief Test of Attention (BTA)

assessed auditory attention and requires no motor response, making it useful for assessing attentional/working memory deficits in subjects with motor/dexterity impairments. The subject listens to progressively longer series of lists containing letters and numbers on an audio tape and after each list is heard, the subject must report either the number of letters or numbers that he or she heard. The total number of correct responses (out of a maximum score of 20) was recorded.

#### Controlled Oral Word Association Test (COWAT)

assessed verbal fluency or the spontaneous production of words beginning with a given letter of the alphabet (letters F, A, S were used) within a limited time period (60 secs.). The total number of words produced (adjusted for education) was recorded.

#### Wisconsin Card Sorting Test (WCST)

assessed set shifting and cognitive flexibility (executive function). A computerized version of the WCST was used. On this test, a stimulus card is presented on the lower portion of the computer screen and the subject is asked to match that card to one of the four cards on the upper portion of the screen. The subject is not told how to make the match but the computer provides feedback as to whether the match is right or wrong. Number of perseverative errors; number of nonperseverative errors; number of categories completed; number of trials to complete first category were recorded.

#### Ruff-Light Trail Learning Test (RULIT)

assessed visuospatial learning and memory. This test does not require good motor control or drawing skills and is designed to minimize or eliminate verbal mediation for successful task completion. Subjects are presented with a stimulus card that contains a complex configuration of circles that are interconnected by lines. The subject begins at the START circle and uses an index finger to trace a line connecting the circles from the START to the END. At each circle (or step) along the trail, there are three to five choices for the next step, but only one choice is correct. The subject is given immediate feedback as to whether their choice was correct, and the subject keeps making choices until the correct choice is made. This process continues through the 15-step trail. Successive trials are administered until the trail is recalled without an error on two consecutive trials. Immediate memory is analyzed by the number of steps correctly completed in Trial 2. Learning is analyzed by the number of trials needed to master the task (total correct)). Long-term (delayed) memory is assessed by having the subject retrace the trail after a 60-minute delay and the number of steps correctly completed was recorded.

#### Conditional Associative Learning Task

assessed learning of arbitrary associations between a set of stimuli and a set of responses. The conditional associative-learning task required subjects to learn, with immediate correction, spatial locations associated with specific stimuli. Four disks and four blank cards are placed are placed in front of the subject. The subject is instructed that each card is associated with only one of these disks and that they need to learn the associations. The experimenter points to a disk and the subject must point to a card and the subject is provided feedback as to whether they have made a correct association. There is a maximum of 3 errors allowed per trial. If 3 errors are made, the administrator shows the subject the correct response. The number of trials to criterion (out of a maximal score of 68) were recorded.

#### Stockings of Cambridge

This test of fronto-executive function assesses planning and problem solving ability and is part of the Cambridge Neuropsychological Test Automated Battery (CANTAB), a computer/touch screen-based cognitive assessment system. In the “plan and move” condition, subjects had to move three colored circles in the lower half of the screen to match a given pattern in the upper half of the screen. The difficulty of the task increased as the number of moves needed to complete the task increased from two to five. Subjects are instructed to wait to begin moving balls until they have planned their moves. In a second condition that controls for motor performance, the “follow” condition, is administered. Subjects are simply required to follow moves made by the computer. By subtracting response times in the “follow” condition from those in the “plan and move” condition, it is possible to separately measure planning and movement times [16]. The number of moves to solution of the problem, the proportion of problems solved in minimum number of moves, the initial thinking time, and the subsequent thinking time were recorded.

#### Spatial Working Memory

This task, also from the CANTAB battery, is a self-ordered spatial search task in which the subject must search for (and find) blue tokens that are hidden inside of colored squares on the screen. The number of items to be searched increases as the task progresses from 2 items to 8 items. Once a blue token had been found within a particular box, that box would not contain another token, as every box was used only once. The test begins with a number of colored boxes shown on the screen and the subject must touch the boxes using a process of elimination, to find one blue ‘token’ in each of the boxes and use them to fill up an empty column on the right hand side of the screen. The number of boxes is gradually increased, until it is necessary to search a total of eight boxes. The color and position of the boxes used are changed from trial to trial to discourage the use of stereotyped search strategies.: For sets of length 4, 6 and 8 boxes, the number of between errors (a subject returns to open a box in which a token has already been found) were assessed.

### Statistical Analysis

Univariate analyses of motor symptoms (M) as measured by the UPDRS part III (motor) scores, cognition (C) as measured by cognitive assessment instruments described above, and demographic characteristics (D) were performed. These included summarizing categorical variables with frequencies and percentages and continuous variables with means, standard deviations, medians, and the interquartile ranges. The distribution of continuous variables was checked using box plots, histograms, and normal probability plots. For demographic variables, comparisons were made between treatment groups using a two-sample t-test with means for continuous variables and using a Chi-square test with percentages for categorical variables. An exact test was used when cell counts were too small for the Chi-square test to be valid. In order to explore relationships between Motor (M) symptoms, Cognition (C), and Demographic (D) variables, Pearson or Spearman correlation coefficients were calculated for each pair of variables. A p-value less than 0.05 was taken to indicate a statistically significant relationship between the corresponding pair of variables interrogated.

## Results

Table 1 shows the demographic characteristics of the sample population (N = 94). This group of PD patients generally had relatively short duration of disease (average 3.0 ± 2.7 years), did not have dementia (based on MMSE criteria), did not have clinical depression (based on BDI-II scores) and were highly educated (average 16.4 ± 3.1 years of education). Relationships between motor symptoms (assessed during a practically defined off period) and demographic features were explored and results showed that all UPDRS subscores (i.e., bradykinesia, posture/balance/gait, speech/facial expression, tremor) with the exception of rigidity were significantly positively correlated with disease duration (Table 2). With the exception of posture/balance/gait, none of the UPDRS subscores were correlated with age at diagnosis and with the exception of rigidity, none of the UPDRS subscores were correlated with MMSE score. None of the UPDRS motor subscores were correlated with years of education, estimated full scale IQ or BDI-II score (Table 2).

**Table 1:** Subject Demographics and Baseline Characteristics.

		PD Subjects (n = 94)
Age (years)	Mean (SD)	59.3 (9.2)
Gender: n (%)	Male	72 (76.6)
	Female	22 (23.4)
Disease duration (years)	Mean (SD)	3.0 (2.7)
	Median (Range)	2.5 (0.4–14.3)
Functional Parameters		
	MMSE score	28.7 (1.2)
	BDI-II score	4.9 (3.9)
	Estimated FS IQ	111.6 (9.8)
	Education (years)	16.4 (3.1)
	Total UPDRS score (Off)	29.8 (10.3)
	UPDRS Motor score (Off)	20.4 (6.8)
	UPDRS ADL score (Off)	8.9 (4.3)
	UPDRS Mentation score (Off)	0.9 (1.0)
Medication Usage		
	Levodopa (# of subjects (percent))	46 (48.9)
	Dopamine Agonist* (# of subjects (percent))	65 (69.1)
	Selegiline (# of subjects (percent))	15 (39.5)
	Other (# of subjects (percent))^	2 (5.2)

Data presented as mean ± SD, unless otherwise noted.

*Dopamine agonists included pramipexole, ropinirole, pergolide and bromocriptine.

^Other medications included amantadine, trihexyphenidyl, and benztropine.

**Table 2:** Correlations Between Off-Period UPDRS Scores and Demographics [r (p value)].

	Bradykinesia	Rigidity	Tremor	Speech/Facial Expression	Posture/Balance/Gait
Age	0.05 (0.62)	0.04 (0.70)	0.01 (0.90)	0.20 (0.05)	**0.28 (< 0.01)**
Disease duration	**0.36 (< 0.01)**	0.09 (0.39)	**0.29 (< 0.01)**	**0.23 (0.03)**	**0.28 (< 0.01)**
MMSE score	0.25 (0.02)	**-0.31 (< 0.01)**	0.07 (0.49)	0.02 (0.86)	-0.09 (0.37)
BDI-II score	-0.01 (0.92)	0.08 (0.44)	-0.05 (0.65)	0.01 (0.89)	0.13 (0.22)
Estimated FS IQ	-0.08 (0.43)	0.01 (0.96)	0.12 (0.25)	-0.08 (0.47)	-0.14 (0.19)
Education	-0.15 (0.14)	-0.06 (0.56)	-0.07 (0.51)	-0.04 (0.73)	-0.10 (0.33)

Statistically significant relationships are shown in bold font.

Relationships between performance on neuropsychological tests and demographic variables (Table 3) and performance on neuropsychological tests and UPDRS components (Table 4) were examined. Auditory attention, assessed with the BTA (number of correct responses), was correlated with MMSE score and FS IQ and negatively correlated with age. Verbal fluency, assessed by performance on the COWAT, was correlated only with MMSE score and FSIQ. Visuospatial learning and memory, as assessed by the RULIT, showed visuospatial learning ability to be positively correlated with MMSE score and negatively correlated with age Immediate and delayed visuospatial memory performance were both negatively correlated with age and immediate memory was also negatively correlated with BDI-II score. Planning and problem solving ability showed relationships with age and education; relationships between cognitive flexibility and age, MMSE score, and education were detected; spatial working memory performance (number of errors made) was correlated with age (i.e., worse performance with increasing age).

**Table 3:** Correlations Between Neuropsychological Performance and Demographic Variables [r (p value)].

	Age	Disease Duration	MMSE score	BDI-II score	Estimated FS IQ	Education
BTA (number correct responses)	**-0.30 (< 0.01)**	-0.13 (0.20)	**0.30 (< 0.01)**	-0.07 (0.48)	**0.30 (< 0.01)**	0.14 (0.19)
COWAT (number of words produced)	-0.07 (0.53)	0.14 (0.19)	**0.31 (< 0.01)**	-0.14 (0.17)	**0.26 (0.01)**	0.08 (0.47)
RULIT (learning: total number correct)	**-0.46 (< 0.01)**	-0.18 (0.09)	**0.22 (0.03)**	-0.07 (0.48)	-0.04 (0.68)	0.14 (0.19)
RULIT (immed. memory: number correct)	**-0.33 (< 0.01)**	-0.17 (0.11)	0.03 (0.77)	**-0.23 (0.03)**	0.10 (0.35)	0.20 (0.06)
RULIT (delayed memory: number correct)	**-0.39 (< 0.01)**	-0.17 (0.10)	0.16 (0.13)	-0.07 (0.51)	0.00 (0.98)	0.15 (0.15)
CAL (trials to criterion)	**0.22 (0.04)**	0.18 (0.08)	-0.13 (0.22)	0.04 (0.67)	-0.05 (0.62)	-0.12 (0.24)
SOC (5 move problems: initial thinking time)	-0.05 (0.61)	-0.16 (0.12)	-0.00 (0.96)	0.00 (0.99)	0.10 (0.34)	0.15 (0.14)
SOC (5 move problems: subsequent thinking time)	**0.25 (0.02)**	-0.02 (0.86)	-0.13 (0.20)	-0.14 (0.17)	-0.03 (0.75)	-0.14 (0.17)
SOC (number solved in minimum moves)	-0.19 (0.06)	0.04 (0.74)	0.15 (0.15)	0.07 (0.52)	-0.07 (0.53)	**0.21 (0.04)**
SOC (number of moves to solve problem)	0.14 (0.19)	-0.13 (0.23)	0.04 (0.74)	-0.07 (0.49)	-0.04 (0.69)	**-0.22 (0.03)**
WCST (number categories completed)	**-0.21 (0.04)**	-0.001 (0.99)	**0.28 (< 0.01)**	0.03 (0.75)	-0.13 (0.22)	**0.25 (0.01)**
WCST (trials to complete first category)	-0.14 (0.18)	-0.001 (0.99)	-**0.43 (< 0.01)**	0.02 (0.88)	**0.18 (0.09)**	-0.12 (0.24)
WCST (number of perseverative errors)	**0.28 (< 0.01)**	0.02 (0.84)	**-0.37 (< 0.01)**	-0.03 (0.76)	**0.22 (0.03)**	**-0.27 (< 0.01)**
WCST (number of nonperseverative errors)	0.10 (0.35)	0.05 (0.64)	-0.15 (0.16)	-0.07 (0.51)	-0.02 (0.83)	**-0.25 (0.01)**
SWM (between search errors 4 boxes)	**0.36 (< 0.01)**	-0.04 (0.74)	**-0.33 (< 0.01)**	-0.08 (0.43)	-0.03 (0.76)	-0.05 (0.63)
SWM (between search errors 6 boxes)	**0.28 (<0.01)**	**0.21 (0.05)**	**-0.43 (<0.01)**	0.09 (0.41)	-0.05 (0.65)	-0.16 (0.14)
SWM (between search errors 8 boxes)	**0.38 (<0.01)**	0.11 (0.29)	-0.14 (0.18)	-0.03 (0.75)	0.04 (0.69)	-0.11 (0.31)

BTA = Brief Test of Attention; RULIT = Ruff-Light Trail Learning Test; CAL = Conditional Associative Learning Test; SOC = Stockings of Cambridge

WCST = Wisconsin Card Sorting Test; SWM = Spatial Working Memory Test. Statistically significant relationships are shown in bold font.

**Table 4:** Correlations Between Neuropsychological Performance and Off UPDRS Motor Scores [r (p value)].

	Bradykinesia	Rigidity	Tremor	Speech/Facial Expression	Posture/Balance/Gait
BTA (number correct responses)	**-0.27 (< 0.01)**	**-0.22 (0.04)**	-0.06 (0.57)	-0.20 (0.05)	**-0.32 (< 0.01)**
COWAT (number of words produced)	-0.14 (0.19)	-0.19 (0.06)	0.12 (0.25)	-0.14 (0.19)	-0.11 (0.30)
RULIT (learning: total number correct)	**-0.22 (0.04)**	-0.10 (0.36)	0.03 (0.75)	**-0.28 (< 0.01)**	**-0.33 (< 0.01)**
RULIT (immed. memory: number correct)	-0.18 (0.08)	-0.1 (0.32)	0.0496 (0.64)	**-0.28 (< 0.01)**	**-0.26 (0.01)**
RULIT (delayed memory: number correct)	**-0.28 (< 0.01)**	-0.18 (0.08)	-0.12 (0.27)	**-0.22 (0.03)**	**-0.34 (< 0.01)**
CAL (trials to criterion)	**0.27 (<0.01)**	0.18 (0.08)	0.03 (0.78)	0.05 (0.62)	**0.39 (< 0.01)**
SOC (5 move problems: initial thinking time)	0.14 (0.20)	-0.00 (0.99)	-0.07 (0.54)	-0.03 (0.78)	0.023 (0.80)
SOC (5 move problems: subsequent thinking time)	0.17 (0.11)	**0.21 (0.05)**	-0.00 (0.95)	0.07 (0.50)	**0.29 (< 0.01)**
SOC (number solved in minimum moves)	-0.02 (0.85)	-0.13 (0.20)	-0.01 (0.92)	**-0.31 (< 0.01)**	**-0.28 (< 0.01)**
SOC (number of moves to solve problem)	0.02 (0.87)	0.09 (0.41)	0.09 (0.37)	0.16 (0.12)	0.20 (0.05)
WCST (number categories completed)	**-0.29 (< 0.01)**	**-0.36 (< 0.01)**	0.06 (0.59)	**-0.31 (< 0.01)**	**-0.24 (0.02)**
WCST (trials to complete first category)	0.20 (0.06)	**0.42 (< 0.01)**	0.03 (0.77)	0.15 (0.14)	0.19 (0.07)
WCST (number of perseverative errors)	0.19 (0.07)	**0.38 (< 0.01)**	-0.07 (0.53)	**0.27 (< 0.01)**	0.18 (0.08)
WCST (number of nonperseverative errors)	**0.28 (< 0.01)**	**0.24 (0.02)**	-0.03 (0.76)	0.20 (0.06)	**0.25 (0.02)**
SWM (between search errors 4 boxes)	0.24 (0.02)	0.13 (0.21)	-0.11 (0.31)	0.18 (0.09)	**0.35 (< 0.01)**
SWM (between search errors 6 boxes)	**0.33(< 0.01)**	0.20 (0.06)	-0.05 (0.63)	**0.33 (< 0.01)**	**0.46 (<0.01)**
SWM (between search errors 8 boxes)	0.17 (0.10)	0.16 (0.12)	-0.15 (0.16)	**0.32 (< 0.01)**	**0.41 (<0.01)**

BTA = Brief Test of Attention; RULIT = Ruff-Light Trail Learning Test; CAL = Conditional Associative Learning Test; SOC = Stockings of Cambridge

WCST = Wisconsin Card Sorting Test; SWM = Spatial Working Memory Test. Statistically significant relationships are shown in bold font.

**Fig 1:**
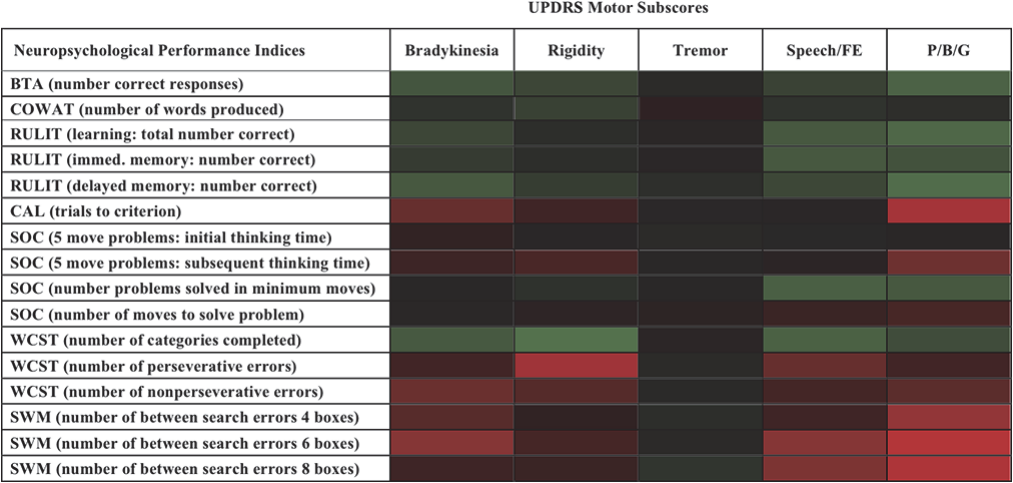
Heat map showing the relationships between cognitive test performance (based on correlation coefficients) and subscores from the Unified Parkinson’s Disease Rating Scale (UPDRS) motor scales. Green denotes a significant negative correlation, red denotes a significant positive correlation and black denotes no significant correlation. The intensity of the color reflects the strength of the correlation.

Performance on the COWAT was not correlated with any UPDRS measures. Brief Test of Attention performance was negatively correlated with bradykinesia, posture/balance/gait/, and rigidity. Conditional associative learning ability (trials to criterion) was correlated only with bradykinesia (i.e., more trials to criterion (worse performance) correlated with higher (worse) bradykinesia scores. Visuospatial learning (RULIT, number correct responses) was negatively correlated with bradykinesia, speech/facial expression, and posture/balance/gait. Immediate visuospatial memory was negatively associated with posture/balance/gait and speech/facial expression while delayed visuospatial memory was also negatively correlated with bradykinesia. Planning and problem solving ability showed minimal relationships with motor function. The number of categories completed on the WCST was negatively correlated with all UPDRS subscores except tremor. The number of errors made during performance of the spatial working memory task at all levels of task difficulty were primarily correlated with posture/balance/gait (i.e., worse performance associated with worse pasture/balance/gait scores). The relationships between neuropsychological test performance (correlation coefficients) and “off” UPDRS motor scores are shown in the heat map in Fig 1.

## Discussion

Non-motor aspects of PD have received increasing attention recently and in particular, it is appreciated that cognitive changes are relatively common in non-demented PD patients [17]. Although the relationships between motor dysfunction and cognition have been investigated previously in PD patients, most studies have examined these relationships in newly diagnosed, drug naïve patients [13] or in more advanced patients in the medicated state, with neuropsychological testing performed in the “on” condition [6]. In the current study, patients requiring anti-PD medication were studied, neuropsychological testing was performed in a practically defined “off” condition, and motor function was assessed in an “off” condition.

For the most part, motor function assessed in the “off” condition correlated primarily with disease duration. Neuropsychological performance primarily was related to age, MMSE score, or education, with disease duration, BDI-II score or FS IQ having little relationship to neuropsychological test results. When the relationships between neuropsychological performance and motor function were examined, it is interesting that tremor was mostly unrelated to neuropsychological outcomes, similar to what has been noted in other studies that examined an earlier PD population [13]. Also, in newly diagnosed, drug naïve PD patients, bradykinesia was negatively associated with tests considered to have executive demands, such as the WCST (categories completed) [13]. In the present study, bradykinesia was also negatively associated with the number of WCST categories completed, reflecting a problem with cognitive flexibility.

Of the UPDRS subscores analyzed, posture/balance/gait was associated with the highest number of adverse cognitive outcomes followed by speech/facial expression, bradykinesia, and rigidity. The relationships between cognitive outcomes and posture/balance/gait scores support the observations that PD patients with a posture instability gait difficulty (PIGD) subtype may have accelerated cognitive decline and an increased risk for developing dementia [18].

No associations were detected between any of the cognitive performance measures and tremor. This lack of association between tremor and cognitive function has been described in other studies [9,10,19] and further suggests that tremor likely results from dysfunction of neural circuits and neurochemical systems that may be distinct from those that underlie cognitive function. In contrast, associations between different aspects of visuospatial function (performance of RULIT task, conditional associative learning, and spatial working memory) and posture/balance/gait were significant, suggesting potentially overlapping processes involved in these functions. In a retrospective analysis of established, non-demented PD patients, it was noted that patients with MCI had higher postural instability and gait disorder subscale scores than cognitively intact patients, suggesting and association of these motor symptoms with increased cognitive dysfunction [20]. Similarly, early stage, drug naïve PD patients were found to have associations between visuospatial function (and episodic memory) and severity of axial symptoms [13]. The findings that these associations span different stages of the disease and are found in early, unmedicated patients as well as in more advanced patients take on particular significance since others have reported that patients whose motor symptoms are prominently characterized by posture and gait disturbances, motor symptoms that are traditionally less responsive to dopaminergic therapy, have increased risk for developing dementia [18,21].

The current study has several potential limitations. The motor function data reported in this paper was the result of clinical assessments and thus relied on clinical judgement of the investigators rather than objective assessments of motor function. However, as described previously by us [22], subjects were observed by two independent raters to improve the precision of this measure. There is also the possibility that some of the neuropsychological measures may have been directly affected by impaired motor function. We think motor disability was unlikely a significant contributing factor to the neuropsychological test results since most tests relied on verbal responses, had no particular timing component that would adversely impact performance, or had built in controls for motor performance. Since this study utilized subjects already receiving anti-PD medications, including levodopa, results obtained during a practically defined “off” period may have been complicated by any potential long duration response to levodopa, and such effects could have contributed to the current findings. However, to the best of our knowledge, long duration effects of levodopa on cognitive function, as assessed in this study, have not been reported. Lastly, because of the number of comparisons made, some of the findings reported here may be not be true associations. We did not correct for multiple comparisons as we wanted to minimize the chance of falsely excluding potentially true relationships, although possibly at the expense of reporting an occasional spurious association. However, we believe that findings related to the lack of any associations of cognitive data with tremor and associations of cognitive data with posture/balance/gait in particular are true as multiple associations within a given cognitive test were found and were all in the same direction. Nevertheless, additional studies may be warranted to confirm our results.

## Conclusions

Our findings suggest that in PD patients who have advanced to requiring anti-PD therapies, there are salient associations between axial signs and different aspects of cognition, and particularly with visuospatial learning and memory suggesting involvement of similar circuits in these functions. Associations between executive functions and bradykinesia also suggest involvement similar circuits in these functions. Age appeared to be the demographic factor most associated with neuropsychological test performance. The more information we gather about possible relationships between demographic factors, cognition, and motor function, the better we might be able to understand the origins of and perhaps predict the course of different cognitive and motor symptoms in PD.
